# MEK Inhibition Induces Canonical WNT Signaling through YAP in *KRAS* Mutated HCT-15 Cells, and a Cancer Preventive FOXO3/FOXM1 Ratio in Combination with TNKS Inhibition

**DOI:** 10.3390/cancers11020164

**Published:** 2019-02-01

**Authors:** Nina Therese Solberg, Maria Melheim, Martin Frank Strand, Petter Angell Olsen, Stefan Krauss

**Affiliations:** 1 Unit for Cell Signaling, Department of Immunology and Transfusion Medicine, Oslo University Hospital, 0372 Oslo, Norway; maria.melheim@rr-research.no (M.M.); petter.angell.olsen@rr-research.no (P.A.O.); Stefan.krauss@rr-research.no (S.K.); 2 Hybrid Technology Hub-Centre of Excellence, Institute of Basic Medical Sciences, Faculty of Medicine, University of Oslo, PO Box 1112 Blindern, 0317 Oslo, Norway; 3 Department of Health Sciences, Kristiania University College, PB 1190 Sentrum, 0107 Oslo, Norway; martinfrank.strand@kristiania.no

**Keywords:** tankyrase, MEK, inhibition, FOXO3, FOXM1, WNT, YAP, colorectal cancer, RNAseq

## Abstract

The majority of colorectal cancers are induced by subsequent mutations in *APC* and *KRAS* genes leading to aberrant activation of both canonical WNT and RAS signaling. However, due to induction of feedback rescue mechanisms some cancers do not respond well to targeted inhibitor treatments. In this study we show that the *APC* and *KRAS* mutant human colorectal cancer cell line HCT-15 induces canonical WNT signaling through YAP in a MEK dependent mechanism. This inductive loop is disrupted with combined tankyrase (TNKS) and MEK inhibition. RNA sequencing analysis suggests that combined TNKS/MEK inhibition induces metabolic stress responses in HCT-15 cells promoting a positive FOXO3/FOXM1 ratio to reduce antioxidative and cryoprotective systems.

## 1. Introduction

Colorectal cancer is the second leading cause of cancer death worldwide (WHO, [1]). About 90% of colorectal cancers (CRCs) are induced by loss-of-function mutations in the *adenomatous polyposis coli* (*APC*) tumor suppressor gene, followed by nuclear accumulation of the canonical WNT signaling effector β-catenin [2]. Subsequent onset of adenoma progression is induced by a secondary oncogenic *KRAS* mutation in 50% of colorectal cancers [3,4,5]. Neither *APC* nor *KRAS* mutations alone induce a colorectal cancer phenotype [6], although *APC* mutations also induce RAS activation through inactivation of glycogen synthase kinase 3β (GSK3β) [7]. The GSK3β containing β-catenin destruction complex is stabilized by both APC and axis inhibition protein 1 and 2 (AXIN1/2) in the absence of canonical WNT signals, promoting proteasomal degradation of both β-catenin (reviewed by [8]) and a subset of RAS proteins [7]. Tankyrase (TNKS) is a central cytoplasmic regulator of the WNT/β-catenin signaling pathway which marks AXIN1/2 for degradation through ADP-ribosylation, and thereby prevents degradation of β-catenin [9,10]. Development of TNKS inhibitors has therefore gained increasing attention as a treatment strategy for WNT induced colorectal cancer.

Due to the extensive crosstalk between major signaling pathways, pathway inhibition in cancer cells commonly experience upregulation of feedback rescue mechanisms in order to survive and maintain their original cell growth potential. The hippo signaling pathway effector YES-associated protein (YAP) has been found to promote resistance to MEK and RAF inhibition in non-small cell lung cancer [11], while TNKS activity protected lung cancer cells from Epidermal Growth Factor Receptor (EGFR) inhibition [12]. Furthermore, MEK inhibition has been identified as a sensitizing factor for TNKS inhibition in *KRAS* mutant CRCs, presumably through inhibition of a feedback rescue mechanism involving Fibroblast Growth Factor Receptor 2 (FGFR2) [13]. Conversely, TNKS inhibition sensitized *KRAS* wild type (WT) CRCs to MEK inhibition [14]. Combining TNKS and RAS/MEK/ERK inhibition is therefore attractive strategies against colorectal cancer although induction of further feedback rescue mechanisms may require extensive combination of inhibitor treatments in order to fully eradicate the cancer [14].

In this study, we used the *KRAS* mutant HCT-15 colorectal cancer cell line as a model system to investigate MEK inhibitor (MEKi) mediated activation of canonical WNT signaling. Taking advantage of the highly specific tankyrase1/2 inhibitor (TNKSi) G007-LK [15], and the highly selective MEKi GDC-0973 [16], we observed a synergistic growth reduction with combined TNKSi/MEKi treatment in HCT-15 cells. In contrast, the *APC* mutant and *KRAS* WT COLO320DM colorectal cancer cell line did not reduce growth or change canonical WNT activity upon treatment with the MEKi, neither alone or in combination with the TNKSi. In order to reveal transcriptional changes that may explain both enhanced canonical WNT signaling with MEKi treatment, and the synergistic growth reduction observed with combined TNKSi/MEKi treatment in HCT-15 cells, we performed RNA sequencing (RNAseq) analysis. Ingenuity pathway analysis (IPA) of RNAseq data suggested the involvement of YAP and FOXM1 in mediating activation of canonical WNT signaling upon MEK inhibition. However, esiRNA mediated knock down (KD) experiments showed that YAP was required for enhanced *AXIN2* transcription, while both YAP and FOXM1 reduction only moderately effected STF/Renilla activation. Furthermore, combined TNKS/MEK inhibition induced a synergistic amount of differentially expressed genes (DEG’s) which were associated with stress responses and cell cycle arrest, inducing a favorable forkhead box protein O3 (FOXO3)/forkhead box protein M1 (FOXM1) ratio to prevent antioxidative and cryoprotective systems.

## 2. Results

### 2.1. MEK Inhibition Sensitizes KRAS Mutant HCT-15 Colorectal Cancer Cells to Tankyrase Inhibition

It has previously been shown that TNKS inhibition sensitizes *KRAS* mutant cancer cells to growth inhibition by MEK inhibitors [13], also in cell lines whose proliferation rate is unaffected by single TNKS inhibitor treatment [14]. To explore the underlying mechanism mediating this effect we initially investigated cell growth in COLO320DM (*APC* mutated/*KRAS* WT) and HCT-15 (*APC* mutated/*KRAS* mutated) colorectal cancer cells under the influence of 1 µM G007-LK (TNKS inhibitor; TNKSi) and/or 1 µM GDC-0973 (MEK inhibitor; MEKi). The biotarget specific responses of TNKSi and MEKi treatments were confirmed by western blot (WB) analysis of TNKS1/2 and phosphorylated MEK1/2 protein levels (Figure S1A,B).

**Figure 1:**
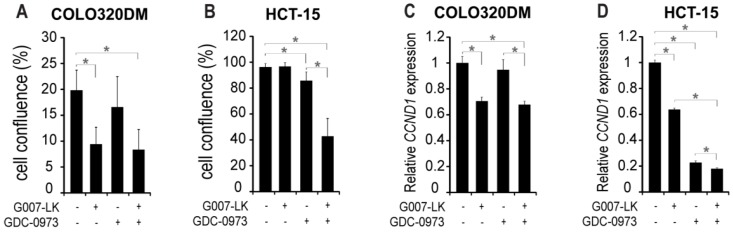
MEK inhibition potentiates HCT-15 cells for growth inhibition by the TNKS inhibitor. Cell confluence at experimental endpoint of the human colorectal cancer cell lines COLO320DM (**A**; 7 days incubation) and HCT-15 (**B**; 6 days incubation) in the presence of inhibitors as indicated. Relative expression levels of *CCND1* were analyzed after 24 h incubation with inhibitors in COLO320DM (**C**) and HCT-15 (**D**) cells. Gene expression was normalized to internal GAPDH levels. Data represents mean relative expression values compared to the DMSO control (± STDEV) of 3 technical replicates. * *p* < 0.05.

TNKS inhibition significantly reduced cell growth by 53% in COLO320DM cells compared to the DMSO control (Figure 1A and Figure S2A), while HCT-15 cells were unaffected (Figure 1B and Figure S2B). MEKi treatment did not significantly influence cell growth in COLO320DM, while in HCT-15 cells MEK inhibition led to a moderate and significant 11% growth reduction. Combined TNKSi/MEKi treatment resulted in similar cell growth effects as single TNKSi treatment in COLO320DM, while in HCT-15 cells the combination synergistically reduced cell growth by 56%.

CyclinD1 (*CCND1*) is required for the cell cycle G1/S transition [17,18], and the transcription level of *CCND1* was investigated after 24 h incubation with inhibitors. In COLO320DM cells *CCND1* transcription was only affected by TNKS inhibition, following the response pattern in cell growth reduction (Figure 1C). In HCT-15 cells, *CCND1* transcription was significantly reduced by both single and combined TNKS and MEK inhibition (Figure 1D), despite no or minor effects on cell growth with single inhibitor treatments. The inconsistency between *CCND1* transcription and cell growth with single inhibitor treatments in HCT-15 cells indicates initiation of CyclinD1 independent rescue mechanisms to maintain normal cell growth. 

### 2.2. MEK Inhibition Induces Canonical WNT Signaling in HCT-15 Cells

**Figure 2:**
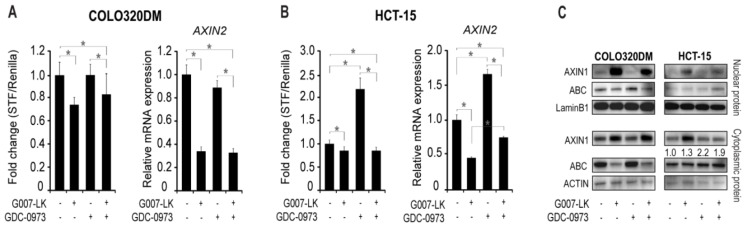
MEK inhibition induces canonical WNT signaling in HCT-15 cells. To reflect canonical WNT signaling upon inhibitor treatments Luciferase reporter assay was conducted on COLO320DM (**A**) and HCT-15 (**B**) cells stably transfected with SuperTop-Flash (STF) and Renilla plasmids. *AXIN2* transcription levels were measured on untransfected cells. Relative gene transcription levels were normalized to internal GAPDH levels. Regulations of AXIN1 and non-phospho (active) β-catenin (ABC) protein levels were investigated by Western blot analysis in untransfected COLO320DM and HCT-15 cells (**C**). LaminB1 and ACTIN were used as loading control for the nuclear and cytoplasmic fraction, respectively. Numbers above ABC blot indicate volume measurements of ABC/ACTIN band intensity, and relative to the DMSO control. Representative graphs and immunoblots from at least 3 independent experiments are shown. Bars in (**A**) and (**B**) indicate mean relative expression values (± STDEV) from at least 3 technical replicates. All assays were performed after 24 h incubation with inhibitors as indicated. * *p* < 0.05.

Canonical WNT signaling is a central driver of cell proliferation, and both COLO320DM and HCT-15 cells display increased canonical WNT activity due to mutations in the *APC* tumor suppressor gene [19]. We therefore wanted to investigate whether the observed responses in cell growth upon single and combined TNKSi/MEKi treatments could be correlated with canonical WNT signaling. COLO320DM and HCT-15 cells stably transfected with the SuperTOP-Flash (STF) reporter gene and control pRL-TK (*Renilla*) plasmid were used, and canonical WNT signaling was quantified after 24 h of treatment with inhibitors. In parallel we also measured the transcription level of the canonical WNT/β-catenin target gene *AXIN2* [20,21]. In COLO320DM cells, TNKSi treatment both alone and in combination with the MEKi, resulted in both reduced STF/Renilla activation (0.7 fold) and *AXIN2* transcription (0.35 fold) compared to the DMSO controls (Figure 2A). In contrast, single MEKi treatment did not influence on either STF/Renilla activity or *AXIN2* mRNA levels, reflecting the observed growth response (Figure 1A).

In HCT-15 cells, TNKSi treatment slightly reduced STF/Renilla activity (0.9 fold) and more strongly reduced *AXIN2* transcription (0.5 fold) compared to the DMSO controls (Figure 2B), despite having no effect on cell growth (Figure 1B). However, in contrast to observations in COLO320DM cells, MEKi treatment enhanced both STF/Renilla activity (more than 2.0 fold) and *AXIN2* transcription (1.7 fold) in HCT-15 cells. Both effects were completely counteracted with combined TNKSi/MEKi treatment. Similar results were obtained in HCT-15 cells with a second independent MEK inhibitor (GDC-0623), thus verifying that the activation of canonical WNT signaling was not due to unspecific GDC-0973 inhibitor effects (Figure S3).

To gain further insight into the cellular responses of TNKS and MEK inhibitor treatments we analyzed the nuclear and cytoplasmic protein levels of central canonical WNT signaling components in COLO320DM and HCT-15 cells. AXIN1 is a structural protein of the cytoplasmic β-catenin destruction complex, and its stability is directly regulated by the catalytic activity of TNKS [9,22]. WB analysis confirmed stabilization of AXIN1 by TNKSi treatment (alone and together with MEKi) in COLO320DM cells, while single MEKi treatment did not affect AXIN1 stability (Figure 2C). Accordingly, both nuclear and cytoplasmic levels of non-phosphorylated (active) β-catenin (ABC) protein were reduced by TNKSi treatment, and unaffected by MEKi treatment.

In contrast, the cytoplasmic protein levels of AXIN1 were not stabilized with combined TNKSi/MEKi treatment in HCT-15 cells, despite increased nuclear AXIN1 protein levels with both single TNKSi and combined TNKSi/MEKi treatments. Furthermore, single TNKSi treatment did not affect ABC levels in HCT-15 cells, while both single MEKi and combined TNKSi/MEKi treatment slightly enhanced both nuclear and cytoplasmic levels of ABC, reflecting the absence of stabilized AXIN1 in the cytoplasm with combined TNKSi/MEKi treatment.

Together these results imply that MEKi treatment induces activation of canonical WNT signaling in HCT-15 cells as an escape mechanism to oppose MEK inhibition, and to maintain normal cell growth. However, this effect is attenuated with combined TNKSi/MEKi treatment, uncoupled from AXIN1 stabilization, and reflected by synergistic cell growth reduction.

### 2.3. RNAseq Analysis Reveals a Positive FOXO3/FOXM1 Ratio with Combined TNKS/MEK Inhibition in HCT-15 Cells

To better understand the global transcriptional changes induced by TNKS and MEK inhibition in HCT-15 cells RNA sequencing (RNAseq) and RNAseq analysis was conducted. Three biological replicates were analyzed after 24 h of treatment with DMSO control, TNKSi and/or MEKi. A principal component analysis (PCA) of the RNAseq data displayed tight clustering of the triplicate samples within each treatment group (Figure S4).

**Figure 3:**
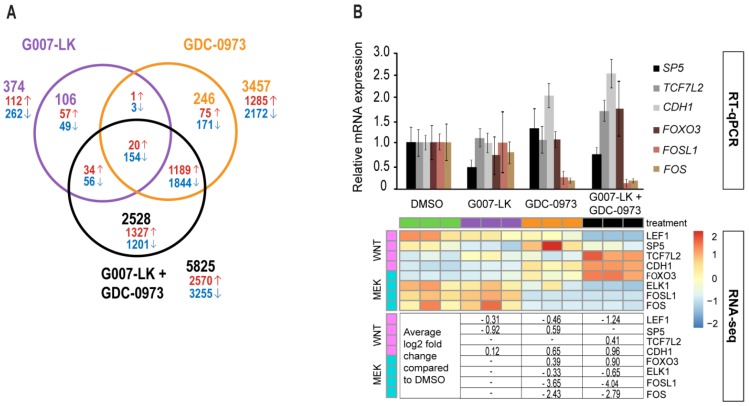
RNAseq analysis of TNKSi and MEKi treated HCT-15 cells. RNAseq count data was analyzed using the count-based statistical method DESeq2. A Venn diagram was created of differentially expressed genes (DEG’s) between the inhibitor treated samples (average of triplicates) compared to the DMSO control (> ±0.5 logfold, padj < 0.1) (**A**). Red numbers indicate upregulated genes, while blue numbers indicate downregulated genes. Purple numbers indicate TNKSi regulated genes, orange numbers indicate MEKi regulated genes, and black numbers indicate combined TNKSi/MEKi regulated genes. The transcriptional changes in a small selection of canonical WNT (*LEF1*, *SP5, TCF7L1* and *CDH1*) and MEK/ERK (*FOXO3, ELK1, FOSL1* and *FOS*) downstream target genes were investigated for their response to inhibitor treatments. The transcription level of *SP5, TCF7L1, CDH1, FOXO3, FOSL1* and *FOS* genes were verified with RT-qPCR on cDNAs created from the same RNA samples as the sequenced RNA (**B**). RT-qPCR data shows mean relative expression values (± STDEV) from the 3 biological replicates, each with 3 technical replicates. Gene transcription levels were normalized to internal GAPDH levels, and are shown as relative to the DMSO control. Individual RNAseq data for the selected genes are visualized with a heatmap, and the average log2 fold changes in gene transcription are indicated in the table below. Only log2 fold changes with a *p*-value < 0.05 are shown. Heatmap Scale bar (red = high, yellow = medium and blue = low) indicates relative differences in log2 fold change within each row.

Differentially expressed genes (DEG’s) identified in a DESeq2 analysis were compared across treatments using a Venn diagram. Single TNKSi treatment induced a relatively low number of 374 DEG’s (106 unique), while single MEKi treatment induced significant transcription changes in 3457 genes (246 unique) compared to the DMSO control (Figure 3A).

Combined TNKSi/MEKi treatment produced 5825 DEG’s, where 2528 of these were uniquely regulated; clearly showing a synergistic regulatory transcriptional effect compared to single inhibitor treatments. Expected TNKSi and MEKi effects were confirmed by investigating canonical WNT (*LEF1, SP5, TCF7L2* and *CDH1*) and MEK/ERK (*FOXO3, ELK1, FOSL1* and *FOS*) downstream target gene transcription regulation in the RNAseq analysis (Figure 3B and Figure S5). Transcription levels of selected genes (WNT; *SP5, TCF7L2* and *CDH1*, MEK/ERK; *FOXO3, FOSL1* and *FOS*) were verified with RT-qPCR analysis on cDNAs created from the same RNA samples (Figure 3B), and RT-qPCR data showed similar patterns as RNAseq data. Both analyses revealed reduced canonical WNT target gene transcription upon TNKS inhibition, and enhanced transcription of the canonical WNT target genes *SP5* (transcription factor) and *CDH1* (E-cadherin) with MEK inhibition. Combined TNKSi/MEKi treatment counteracted the MEKi induced transcription of *SP5*, while *TCF7L2* (TCF4) transcription was induced only when the inhibitors were combined. MEK/ERK target genes were only regulated by the MEKi, although MEK inhibition potentiated HCT-15 cells to TNKS inhibition by further reducing MEK/ERK target gene transcription with combined TNKSi/MEKi treatment.

**Figure 4:**
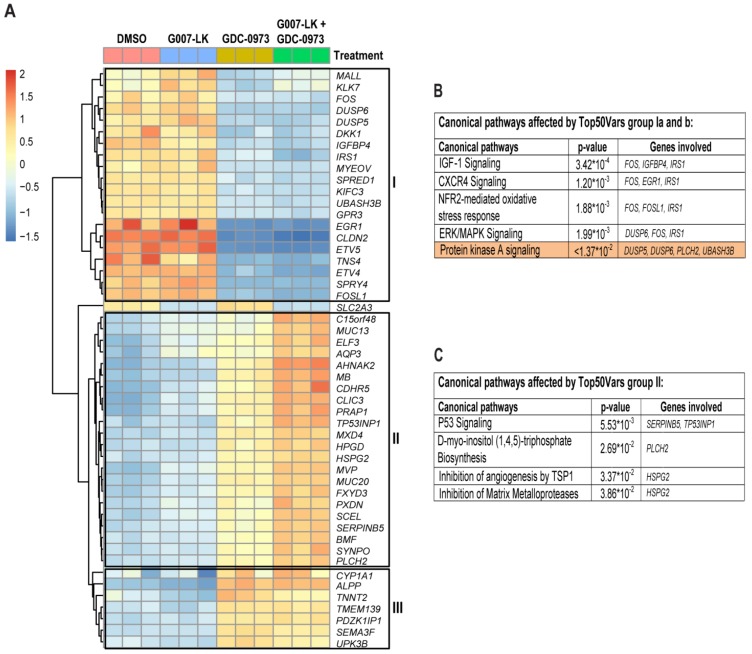
Characterization of the 50 most regulated genes regardless of inhibitor treatment in HCT-15 cells A heatmap of the 50 most regulated genes across the different treatments compared to the DMSO control was created (**A**). 3 distinct groups of regulations were revealed. One group (I) consisting of 20 genes was mainly downregulated by the MEKi, with minor additional changes in combination with TNKS inhibition. A second group (II) consisting of 22 genes was moderately upregulated by the TNKSi, more clearly upregulated by the MEKi, and additively upregulated by combined TNKSi/MEKi treatment. The third group (group III) consisting of 7 genes was upregulated by the MEK inhibitor, with minor additional changes in combination with TNKS inhibition. *SLC2A3* (GLUT3) gene transcription was downregulated by the TNKSi, with minor additional changes in combination with the MEKi. Canonical pathways predicted by the Ingenuity pathway analysis (IPA) core analysis to be regulated by group I and III is indicated in (**B**). With an applied cutoff at (*z*-score > ±2, padj < 0.01), only the Protein Kinase A pathway was significantly affected (highlighted). The canonical pathways predicted by the IPA to be regulated by genes in group II is shown in (**C**). Heatmap Scale bar (red = high, yellow = medium and blue = low) indicates relative differences in log2 fold change within each row. Individual regulations in biological replicates are shown for all treatment groups.

Next, a heatmap of the 50 most regulated genes compared to the DMSO control, regardless of inhibitor treatment, was created (Figure 4A and Tables S1 and S2). The heatmap revealed 3 distinct groups of transcriptional regulation. One group (20 genes) was downregulated by the MEKi compared to the DMSO control, with minor additional changes in combination with TNKS inhibition (group I). Within this group we observed the *ETV4*, *ETV5* and *MYEOV* genes which have been shown to promote resistance to MAP Kinase inhibitors [23] as well as promoting cancer cell proliferation, invasion and migration [24]. The second group consisting of 22 genes (group II) was mildly upregulated by single TNKS and MEK inhibition, with additive effects with combined TNKSi/MEKi treatment. Among these genes we observed *CLIC3*, *PRAP1* and *AHNAK2*, which have been shown to promote cancer progression of gallbladder carcinoma [25], chemotherapeutic drug resistance in colorectal cancer [26] and epithelial to mesenchymal transition in clear cell renal cell carcinoma [27], respectively. The third group (goup III) consists of 7 genes which were upregulated by the MEKi, and with minor changes by the TNKSi. Ingenuity pathway analysis (IPA) software was used to investigate canonical pathways affected by genes in group I and III (mostly affected by the MEKi).

IPA predicted *CTNNB1* (β-catenin) as the top upstream regulator of these genes (activation *z*-score; −2.183, Table S3), affecting “IGF-1 signaling”, “CXCR4 signaling”, “NRF2 mediated oxidative stress response” and “ERK/MAPK signaling” (Figure 4B). With a pre-defined cutoff (*z*-score > ±2, padj < 0.01) only the Protein Kinase A (PKA) pathway was significantly affected (upregulated). For the genes in group II, the IPA software predicted *TP53* (p53) as the top upstream regulator (activation *z*-score; 1.148, Table S4), affecting “p53 signaling”, “D-Myoinositol (1,4,5) Trisphosphate Biosynthesis”, “Inhibition of angiogenesis by TSP1”, and “inhibition of matrix metalloprotease” (Figure 4C). Finally, only *SLC2A3* gene transcription (GLUT3 - involved in glucose transport) was mainly affected (downregulated) by the TNKSi, with minor additional changes in combination with MEK inhibition. Together these results show that the 50 most regulated genes were mostly influenced by the MEKi to affect both metabolic and oxidative stress responses, as well as growth factor signaling pathways, primarily affecting molecular and cellular functions involving cell growth, development and survival (Figure S6).

Next, we wanted to understand the underlying mechanism of the individual treatments in relation to their effects on cell growth. The IPA software was therefore used to characterize the canonical pathways and biological functions significantly associated with single inhibitor induced DEG’s. At the selected dose, single TNKSi treatment induced only minor transcriptional changes in HCT-15 cells (Figure 3A). With a mild cutoff at log fold change (lfc) > ±0.5, padj < 0.1 [data input], *z*-score > ±2 (analysis output), the IPA software associated TNKSi induced DEG’s with enhanced “cholesterol biosynthesis”, enhanced “ErbB signaling”, enhanced “glioma invasiveness signaling” and enhanced “NFR2-mediated oxidative stress response” (Figure S7A and Tables S5 and S6). On the other hand, MEK inhibition induced more significant transcriptional changes at the selected inhibitor dose which the IPA software (cutoff at lfc > ±2, padj < 0.01 [data input], *z*-score > ±0.4 [analysis output]) associated with reduced “ERK/MAPK signaling”, induced “PKA signaling” and induced “osteoarthritis pathway” (Figure S7B and Table S7). Inhibited *STAT3* and *CTNNB1* were predicted as the main upstream regulators of these events (Table S8). Furthermore, both TNKSi and MEKi induced DEG’s were associated with molecular and cellular functions like cell growth and proliferation, cellular development, and cell death and survival (Figure S7C,D).

**Figure 5:**
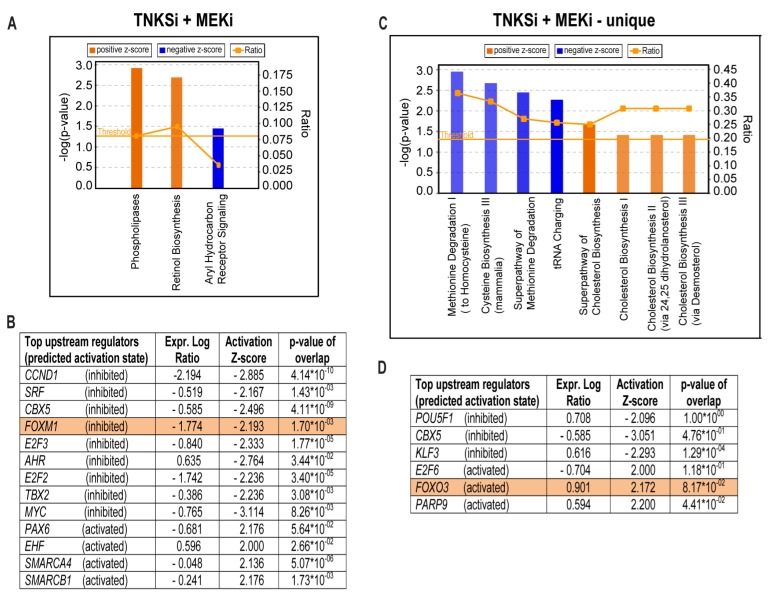
Combined TNKSi/MEKi treatment induces a positive *FOXO3*/*FOXM1* ratio. The IPA software core analysis (lfc > ±2, padj < 0.01 [data input], *z*-score > ±2 [analysis output]) was used to predict the canonical pathways (**A**) and upstream regulators (**B**) associated with DEG’s induced by combined TNKSi/MEKi treatment. The IPA software also predicted canonical pathways (**C**) and upstream regulators (**D**) associated with DEG’s uniquely induced by combined TNKSi/MEKi treatment. Only predicted upstream regulators with a measured expression log ratio are listed in (**B**) and (**D**). These analyses predict that a positive FOXO3/FOXM1 ratio is induced by combined TNKS7/MEKi treatment (highlighted). Inconsistency between predicted activation state and the measured transcriptional regulation may be related to the corresponding protein activity rather than the gene transcription.

Due to the large amount of significant DEG’s induced by combined TNKSi/MEKi treatment (Figure 3A and Figure S8) we performed IPA analysis using a more stringent cutoff (lfc > ±2, padj < 0.01 [data input], *z*-score > ±2 [analysis output]), which associated these DEG’s with upregulation of “phospholipases”, upregulation of “retinol biosynthesis”, and downregulation of “aryl hydrocarbon receptor signaling” (Figure 5A and Table S9). These DEG’s were further predicted to affect biological functions like cellular development, cell growth and proliferation, cell death and survival, cellular movement, and cell cycle (Figure S9A). Inhibition of cell cycle regulators like *CCND1*, *SRF*, *FOXM1* and *MYC*, and upregulation of transcriptional regulators like *PAX6*, *EHF*, *SMARCA4* and *SMARCB1* was predicted as main upstream regulators of these processes (Figure 5B and Table S10). However, we also observed an enhancement (*z*-score = 0.378, *p*-value 0.012) in ERK/MAPK signaling which suggests an induction of a feedback rescue mechanism. This is in compliance with enhanced EGFR activity previously observed with combined TNKSi/MEKi treatment in HCT-15 cells [14], and may partly account for the sustained cell growth (Figure 1B).

Finally, we investigated the 2528 DEG’s (Figure 3A) uniquely regulated by combined TNKSi/MEKi treatment. According to the IPA software (lfc > ±2, padj < 0.01 [data input], *z*-score > ±2 [analysis output]) these genes were predicted to significantly downregulate “methionine degradation”, downregulate “cysteine biosynthesis”, downregulate “tRNA charging”, and upregulate “cholesterol biosynthesis” (Figure 5C and Table S11). Furthermore, these DEG’s were predicted to affect general gene expression, DNA recombination and repair, RNA post-transcriptional modification, and cellular assembly and organization (Figure S9B). Inhibition of *POU5F1* (OCT-4), *CBX5*, and *KLF3*, and activation of *E2F6*, *FOXO3* and *PARP9* was predicted as main upstream regulators of these processes (Figure 5D and Table S12). Together these observations suggest that TNKSi/MEKi induced DEG’s initiate several stress responses and rescue mechanisms in HCT-15 cells which target basic cellular functions like cell renewal and survival. Furthermore, predicted activation of *FOXO3* and inhibition of *FOXM1* provides a positive FOXO3/FOXM1 ratio which is considered to be favorable, since these factors are crucial in various aspects of cancer progression [28,29,30]. In particular, FOXO3 has been shown to both inactivate *FOXM1* at the transcriptional level and to compete for the same target genes as FOXM1 [31,32].

### 2.4. RNAseq Analysis Suggests YAP or FOXM1 Mediated Induction of Canonical WNT Signaling

Despite reduced canonical WNT signaling (Figure 2B) the growth rate of HCT-15 cells was not affected by single TNKSi treatment (Figure 1C). A detailed evaluation of the DESeq2 and IPA analyses revealed that TNKS inhibition downregulated transcription of canonical WNT pathway mediators like *Frizled, WNT* and *AXIN2* (Figures S10 and S18, and Table S13), supporting reduced canonical WNT signaling. Additionally, TNKS inhibition induced transcription of *RAS* and downstream Protein kinase B (AKT) effectors (Figures S11 and S18), which indicates initiation of a feedback rescue mechanism through AKT to maintain normal cell growth in response to inhibition of canonical WNT signaling.

Furthermore, MEKi treatment reduced transcription of several genes involved in cell cycle regulation (Figure S12 and Table S14), as well as enhanced transcription of *RAS* and *ERK1/2* (Figures S13 and S19), which could be due to the previously reported MEKi induced activation of EGFR in HCT-15 cells [14]. These contradictory transcriptional regulations support the mild response in growth reduction upon MEK inhibition in HCT-15 cells. MEKi treatment also reduced transcription of the canonical WNT inhibitor *DKK1* and the transcriptional co-repressor *Groucho*, while enhancing transcription of canonical WNT signaling activators like *SOX* and *TCF/LEF*s (Figures S14 and S19), supporting enhanced canonical WNT signaling (Figure 2B). Furthermore, on the transcriptional level we observed that MEKi induced reactivation of WNT also enhanced transcription of the canonical WNT/YAP target genes *ASCL2, BCL2L1* and *BIRC5* [33,34,35,36] (Figure S15A–C), which was further supported by enhanced transcription of several genes in the hippo/YAP signaling pathway (Figure S16). These effects were counteracted with combined TNKSi/MEKi treatment (Figures S15A–C, S17 and S20, and Tables S10 and S15). Single MEKi treatment also reduced transcription of the YAP target gene *FOXM1* [37], followed by reduced transcription of FOXM1 target genes, which was further reduced with combined TNKSi/MEKi treatment (Figure S15D,E). Following reduced transcription of *FOXM1* we also observed increased transcription of canonical WNT target genes like *GATA6, WNT7B, SOX4* and *TCF4* (Figure S15F), previously shown to be upregulated in the absence of FOXM1 [38].

These results are in agreement with the MEKi induced activation of canonical WNT activity (Figure 2B), as well as with predicted effects of WNT mediated activation of nuclear YAP in the intestinal crypt [39], and suggest a mechanism involving YAP and/or FOXM1. Thus, we proceeded to investigate whether YAP and/or FOXM1 are involved in a MEKi induced rescue mechanism.

### 2.5. Feedback Activation of YAP is Responsible for Enhanced AXIN2 Transcription upon MEK Inhibition in HCT-15 Cells

Previous studies have demonstrated the induction of YAP activity upon loss of the tumor suppressor *APC* in colon cancer [39,40], or as a mechanism to bypass oncogenic *KRAS* addiction in pancreatic cancer cells [36]. *FOXM1* is one of the YAP target genes [37], and is a driver of cell proliferation, chemo resistance and cancer progression [28]. FOXM1 has also been shown to promote nuclear localization of β-catenin and induction of canonical WNT target gene transcription during glioma tumorigenesis [41,42]. Based on these observations, and the above mentioned RNAseq results, we explored whether regulation of YAP/FOXM1 signaling were responsible for the MEKi induced elevation in canonical WNT signaling in HCT-15 cells. In addition, we also explored whether the canonical WNT mediator β-catenin was involved.

**Figure 6:**
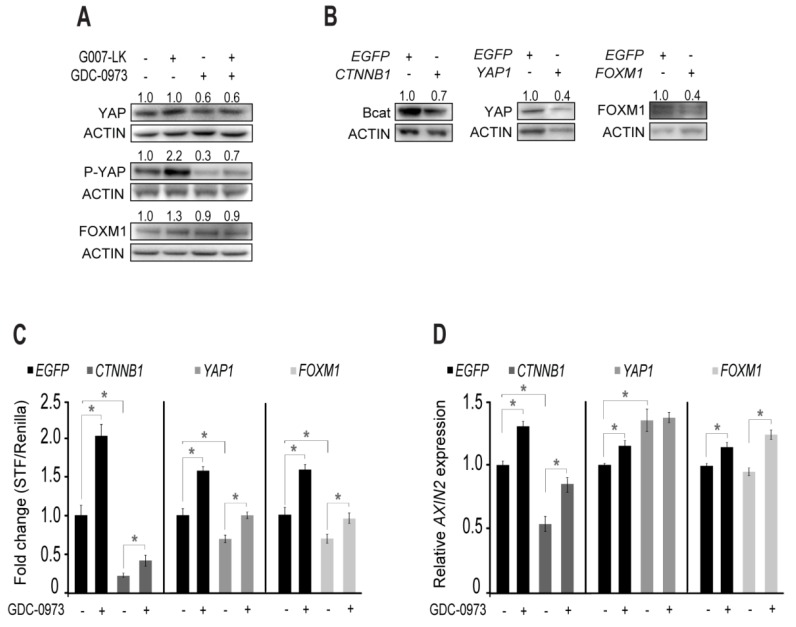
Feedback activation of YAP is responsible for enhanced *AXIN2* transcription upon MEK inhibition in HCT-15 cells. Regulations of YAP, P-ser127 YAP (P-YAP) and FOXM1 protein levels in total cell extracts from HCT-15 cells after 24 h incubation with inhibitors as indicated (**A**). Numbers above blots indicate volume measurements of Protein/ACTIN band intensity, and relative to the DMSO control. Western blot analysis showing total β-catenin (Bcat), YAP and FOXM1 protein levels levels after esiRNA mediated knock down (KD) of β-catenin, YAP and FOXM1 (**B**). Individual KD data is compared to their respective EGFP controls. Luciferase reporter assay (**C**) and *AXIN2* transcription levels (**D**) were measured on esiRNA mediated KD cells after 24 h of treatment with DMSO control media or MEK inhibitor containing (1 µM GDC-0973) media as indicated. All KD related assays were conducted on HCT-15 cells stably transfected with SuperTop-Flash (STF) and Renilla plasmids. Gene transcription levels were normalized to internal GAPDH levels prior to their relative comparison to the DMSO control. All experiments shown are performed in parallel and are representative graphs/immunoblots from at least 3 independent experiments. Bars in (**B**–**D**) indicate mean relative expression values (± STDEV) from at least 3 technical replicates. * *p* < 0.05.

The protein level of total YAP was unaffected by TNKS inhibition while moderately downregulated with both single and combined MEK inhibition (Figure 6A), reflecting *YAP1* transcription levels (Figure S15B,G). In contrast, YAP was strongly inactivated (P-Ser127-YAP) by TNKS inhibition (Figure 6A), while MEKi treatment reduced inactivation of YAP compared to the DMSO control. Combined TNKSi/MEKi treatment partially counteracted the TNKSi mediated inactivation of YAP. Furthermore, FOXM1 protein level was slightly enhanced by TNKS inhibition, while with MEK inhibition the FOXM1 protein level remained unaffected (Figure 6A).

To investigate whether β-catenin, YAP or FOXM1 are responsible for MEKi induced canonical WNT signaling we performed esiRNA mediated knock down (KD) of *CTNNB1*, *YAP1* and *FOXM1* transcripts in HCT-15 cells. This resulted in 30% (β-catenin), 60% (YAP) and 60% (FOXM1) reduction in the corresponding protein levels (Figure 6B). Next, we measured whether reduction in β-catenin, YAP and FOXM1 proteins affected the potential of the MEKi to induce canonical WNT signaling. Compared to their respective EGFP esiRNA controls we observed 77% (*CTNNB1*), 30% (*YAP1*) and 30% (*FOXM1*) reduction in STF/Renilla activity with DMSO control treatment (Figure 6C), suggesting that all three proteins were involved in mediating canonical WNT signaling in HCT-15 cells. However, upon MEKi treatment, STF/Renilla activity was similarly induced in both EGFP control cells (2.0 fold) and *CTNNB1* KD cells (1.9 fold) (Figure 6C). Upon *YAP1* and *FOXM1* KD, MEKi treatment induced STF/Renilla activity 1.4 fold, compared to 1.6 fold in the corresponding EGFP control cells. Together, this shows that β-catenin, YAP and FOXM1 are involved in mediating canonical WNT signaling in HCT-15 cells, while both YAP and FOXM1 was partially responsible for the MEKi induced canonical WNT signaling.

Since MEK inhibition also induced *AXIN2* transcription in HCT-15 cells (Figure 2B), we investigated whether reduction in β-catenin, YAP or FOXM1 proteins affected *AXIN2* transcription. Reduction in β-catenin protein reduced transcription of *AXIN2* by 49% compared to EGFP control with DMSO treatment, while reduced YAP protein significantly enhanced transcription of *AXIN2* (1.3 fold compared to EGFP) (Figure 6D). Reduction in FOXM1 protein did not change *AXIN2* transcription. Furthermore, MEKi treatment significantly enhanced *AXIN2* transcription upon *EGFP* control KD (1.3 fold), *CTNNB1* KD (1.6 fold) and *FOXM1* KD (1.3 fold). However, MEKi treatment did not alter the transcription level of *AXIN2* in YAP reduced HCT-15 cells. We therefore conclude that the MEKi induced transcription of *AXIN2* is mediated by YAP in HCT-15 cells, although YAP also partly restrains *AXIN2* transcription in the absence of MEKi treatment.

We therefore conclude that HCT-15 cells induce canonical WNT signaling through YAP (*AXIN2* transcription) and YAP/FOXM1 (STF/Renilla activity) as a feedback rescue mechanism to maintain proliferation and survival upon MEK inhibition. Since TNKS inhibition restrains both canonical WNT [43] and hippo/YAP [44] activity, combining MEK inhibition with TNKS inhibition disrupts the feedback rescue mechanism induced by single agent MEK inhibition.

## 3. Discussion

Abnormal activation of canonical WNT signaling promotes nuclear translocation of β-catenin and induces transcription of genes which initiate and maintain colon cancers [3,45]. In this study we observed that MEK inhibition further induced canonical WNT signaling in *APC* and *KRAS* mutant HCT-15 colorectal cancer cells, but not in *APC* mutant/*KRAS* WT COLO320DM cells (Figure 2). In HCT-15 cells, MEK inhibition induced elevated β-catenin protein level, increased STF/Renilla activity and subsequently enhanced transcription of the canonical WNT target gene *AXIN2*. RNAseq analysis supported these findings showing induced transcription of typical canonical WNT target genes upon MEK inhibition, while transcription of canonical WNT inhibitors was reduced (Figures S14, S15F and S19). Enhanced β-catenin protein level mediated by the MEKi was initially proposed as the obvious candidate mediating these typical canonical WNT responses. However, esiRNA mediated KD experiments revealed that enhanced *AXIN2* transcription was mediated by YAP, while both YAP and FOXM1 partially influenced on STF/Renilla signaling (Figure 6). MEK targeted cancer therapies were previously shown to induce YAP activity as a survival mechanism [36,46], where nuclear active YAP promotes tumorigenesis by inducing expression of the anti-apoptotic protein BCL-xL (*BCL2L1*) [46]. Furthermore, YAP has also been shown to induce *AXIN2* transcription in muscle fibers [47], which is in agreement with MEKi induced *AXIN2* transcription in HCT-15 cells. However, YAP has also been observed to restrain AXIN2 transcription in mouse embryonic stem cells [39] and MCF-10 breast cancer cells [48], which is in line with the mild induction of *AXIN2* transcription in HCT-15 cells upon *YAP1* KD (Figure 6D). It is therefore clear that in the absence of treatment YAP restrain *AXIN2* transcription in HCT-15 cells, while the MEKi induces *AXIN2* transcription through a parallel mechanism which is also dependent on YAP. Oppositely, TNKS inhibition reduces YAP activity in HCT-15 cells, probably through sequestration of YAP in the β-catenin destruction complex [39], which may further explain the attenuation of the MEKi induced WNT effects with combined TNKSi/MEKi treatment. We therefore conclude that HCT-15 cells induce canonical WNT signaling through YAP as a feedback rescue mechanism to maintain proliferation and survival upon MEK inhibition. Combining MEK inhibition with TNKS inhibition is therefore an effective tool to disrupt this effect.

However, while the observed increase in STF/Renilla activity and *AXIN2* transcription upon MEK inhibition were attenuated in combination with the TNKSi, the β-catenin protein level remained elevated compared to the DMSO control. This could be linked to the absence of AXIN1 stabilization observed when the TNKSi was combined with the MEKi (Figure 2C). MEK inhibition has previously been shown to reduce the level of AXIN1 protein in melanoma cells [49], although the exact mechanism of ERK/MAPK regulation of AXIN1 needs further investigation. Reduced level of AXIN1 may subsequently result in a compromised β-catenin destruction complex, followed by enhanced β-catenin protein levels and nuclear accumulation of both β-catenin and YAP proteins [39].

**Figure 7:**
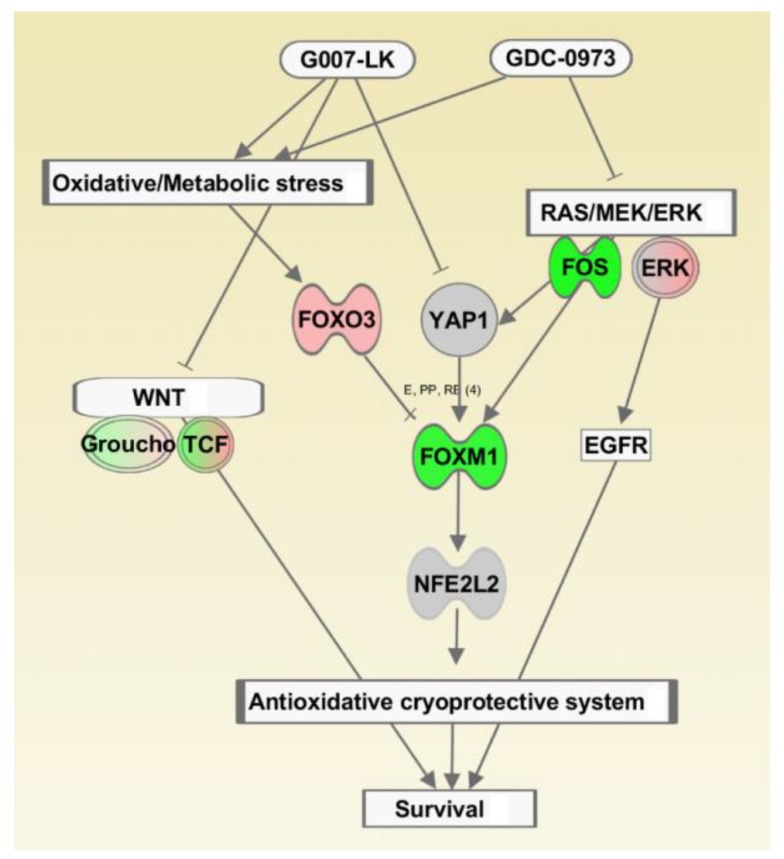
Proposed model of drug induced oxidative stress responses in HCT-15 cells. IPA generated model of drug induced stress responses in HCT-15 cells with an overlay of DEG’s (log2 fold > 0.5, padj < 0.1) produced by combined TNKS/MEK inhibition. Combined TNKSi/MEKi treatment promoted a positive FOXO3/FOXM1 ratio which subsequently downregulated the NRF2 modulated antioxidative and cryoprotective systems. Together with downregulation of canonical WNT and YAP activity the combined TNKSi/MEKi treatment also synergistically reduced cell survival by inducing molecular characteristics of cell cycle arrest and apoptosis. However, a feedback rescue mechanism mediated by ERK on EGFR is proposed to maintain cell growth of the most treatment resistant cells.

Enhanced YAP activity upon MEK inhibition in HCT-15 cells was observed by WB analysis (Figure 6A), as well as proposed by the RNAseq analysis through enhanced transcription of the canonical WNT/YAP target genes *ASCL2, BCL2L1* and *BIRC5* (Figure S15A–C). Subsequently, the IPA also predicted that combined TNKSi/MEKi treatment induced several stress response pathways in HCT-15 cells, including “NRF2 mediated oxidative stress response” (Figure 4 and Figure 5 and Figure S7), which has been linked to resistance to oxidants and to promote aggressive proliferation of cancer cells [50]. Previous reports have proposed a bimodal regulation between NRF2 (*NFE2L2*) and YAP where silencing of NRF2 has been shown to inhibit *YAP1* transcription, while active YAP induced transcription of *FOXM1* in combination with its co-factor TEAD [37]. Finally, FOXM1 was subsequently shown to induce *NFE2L2* transcription [51]. In HCT-15 cells we observed reduced transcription of *YAP1, TEAD, FOXM1* and *NFE2L2* upon MEK inhibition (Figure S15B,D,H), which were further reduced in combination with the TNKSi. Together this proposes a connection between YAP and NRF2 mediated stress responses in HCT-15 cells (Figure 7). Furthermore, NRF2 mediated stress response has also been shown to promote PKA signaling in cancer cells in order to mediate resistance to glucose starvation [52]. Both PKA signaling and *SLC2A3* (GLUT3) transcription were significantly regulated upon treatment in HCT-15 cells (Figure 4).

Metabolic stress and increase in reactive oxygen species (ROS) have been shown to induce FOXO3 activation, enhance transcription of pro-apoptotic genes and induce differentiation [53]. RNAseq data analysis predicted a significant change in the FOXO3/FOXM1 ratio, and highlighted both FOXO3 (activated) and FOXM1 (inhibited) as top upstream regulators of DEG’s that are induced with combined TNKSi/MEKi treatment (Figure 5). FOXO3 and FOXM1 are paralogs belonging to the O subclass of the forkhead family of transcription factors, and compete for the same target genes [31,32]. However, while FOXO3 activity is thought to promote apoptosis and cell cycle arrest, FOXM1 promotes cell proliferation, survival, DNA repair and drug resistance (reviewed by [28]). In HCT-15 cells, reduced transcription of cell cycle mediating genes (*CCND1*, *PCNA*, *CDK2*, *CDKN1B*; Figure S15D,H,I), and reduced transcription of anti-apoptotic genes (*BMI1*, *BIRC5* and *BCL2*; Figure S15D,J), support a cancer suppressive and favorable FOXO3/FOXM1 ratio [28] upon combined TNKSi/MEKi treatment. This is further reflected by a synergistic growth reduction compared to single inhibitor treatments in HCT-15 cells.

## 4. Materials and Methods

### 4.1. Cell Lines

The human colorectal cancer cell lines COLO320DM (ATCC^®^ CCL-220™) and HCT-15 (ATCC^®^ CCL-225™) were grown according to the manufacturers protocol in RPMI-1640 with L-glutamine and sodium bicarbonate medium (Sigma-Aldrich, St Louis, MO, USA, R8758), supplemented with 10% fetal bovine serum (FBS) (Gibco Products International, Big Cabin, OK, USA, 10270106), and 1% Penicillin/Streptomycin (Pen/Strep) (Sigma-Aldrich, P4333) at 37 °C in a humidified atmosphere containing 5% CO_2_. Cells were routinely tested for *Mycoplasma* using the MycoAlert Mycoplasma detection kit (Lonza Group Ltd, Basel, Switzerland).

### 4.2. Inhibitors

Inhibitors were dissolved in dimethyl sulfoxide (DMSO; Sigma-Aldrich, 0.02%), which was also used as vehicle control. The following inhibitors were used: G007-LK (ChemRoyal Inc., Atlanta, GA, USA, 1 µM), GDC-0973 (MedChem Express, Sollentuna Sweden, 1 µM) and GDC-0623 (MedChem Express, 1 µM).

### 4.3. Proliferation Analysis

A total of 1000 COLO320DM and HCT-15 cells were plated in six parallels per treatment in a 96-well plate (Nunc^TM^,Thermo Fischer Scientific, Boston, MA, USA). The following day, culture media was replaced with vehicle or inhibitor containing media. Cell confluence was quantified with the IncuCyte live-cell analysis system (Essen BioScience, Ann Arbor, MI, USA). Images were captured every second hour to monitor proliferation and results were retrieved through corresponding IncuCyte 2011A software (Essen BioScience), and exported to Excel for further analysis and graph design.

### 4.4. SuperTopFlash (STF) Assay

The COLO320DM-STF and HCT-15-STF cell lines have been described previously [43]. A total of 15,000 cells were plated per well in a 96-well plate (Nunc^TM^). The following day, culture media was replaced with vehicle or inhibitor containing media. After 24 h of treatment luciferase activity was measured using the Dual-Luciferase Reporter Assay 1000 kit (Promega, Fitchburg, WI, USA) according to the manufacturer’s protocol, and with the GloMax®-Multi Detection System (Promega). Results were retrieved through corresponding GloMax®-Multi + Detection System Instinct^TM^ Software (Promega), and exported to Excel for further analysis and graph design. All STF Assays were performed with at least 3 biological replicates, each with at least 3 technical replicates.

### 4.5. RNA Extraction and Quantitative Real Time PCR (RT-qPCR)

Total RNA was isolated after 24 h of treatment using a GenEluteTM Mammalian Total RNA Miniprep Kit (Sigma Aldrich) following the manufacturer’s instructions. cDNA was synthesized from 1 µg total RNA using a SuperScript® VILO cDNA synthesis Kit (Life Technologies, Carlsbad, CA, USA) following the manufacturer’s instructions. Prior to RNA sequencing, samples were treated with DNaseI according to manufacturer’s instructions (Sigma Aldrich). RT-qPCR was carried out using a TaqMan Gene expression Mastermix (Life Technologies, 4369510) and ViiA7 (Applied Biosystems, Foster City, CA, USA). The amplification protocol was initiated with 2 min at 50 °C followed by denaturation for 10 min at 95 °C, then 40 cycles with denaturation for 15 s at 95 °C, annealing of TaqMan probes and amplification at 60 °C for 1 min. All RT-qPCR analyses were performed with 3 biological replicates, each with 3 technical replicates. Gene expressions are normalized to the *GAPDH* internal control, and bars indicate fold change relative to DMSO control.

The following probes were used (all from Life Technologies): AXIN2; Hs00610344_m1, 4351370. CCND1; Hs00765553_m1, 4331182.CTNNB1; Hs00991810_g1, 4351372. FOXM1; Hs01073586_m1, 4453320. SP5; Hs01370227_mH, 4453320. ASCL2; Hs00270888_s1, 4351370. BIRC5; Hs04194392_s1, 4448892. TCF7L2; Hs01009044_m1, 4448892. CDH1; Hs01023894_m1, 4453320. FOXO3; Hs04195365_s1, 4448892. FOSL1; Hs04187685_m1, 4448892. FOS; Hs01119266_g1, 4448892. YAP1; Hs00371735_m1, 4453320. GAPDH; Hs02758991_g1, 4351368.

### 4.6. esiRNA Mediated Knock Down

800,000 HCT-15 cells were plated per well in a 6-well dish. The following day the culture media was exchanged with media without antibiotics and transfected with either 50 nM EGFP esiRNA (EHUEGFP, Sigma-Aldrich,), 50 nM CTNNB1 (EHU139421, Sigma-Aldrich), 50 nM YAP1 (EHU113021, Sigma-Aldrich) or 50 nM FOXM1 esiRNA (EHU124431, Sigma-Aldrich), using Lipofectamine RNAiMax Transfection Reagent (ThermoFisher, Boston, MA, USA, 13778075). The following day 15,000 cells were seeded in 96-well plates for luciferase assay, 150,000 cells were seeded in 12-well plates for RNA isolation, and 400,000 cells in 6-well plates for protein analysis. Total protein extracts were monitored for β-catenin, YAP and FOXM1 reduction 72 h post transfection. All esiRNA analyses were performed with three biological replicates.

### 4.7. RNA Sequencing

Total RNA was isolated using GenElute^TM^ Mammalian Total RNA Miniprep Kit (Sigma Aldrich), followed by DNaseI (Sigma Aldrich) treatment for 15 min, according to the manufacturer’s instructions. Sequencing libraries were generated from three biological replicates of cultured HCT-15 cells treated with DMSO (0.02%), G007-LK (1 µM), GDC-0973 (1 µM) or G007-LK and GDC-0973 for 24 h using the Illumina TruSeq stranded mRNA kit (Illumina Inc., San Diego CA, USA). The libraries were subsequently sequenced on the HiSeq2500 (Illumina Inc.) at the Genomics Core Facility Oslo (Oslo University Hospital, Norway). Reads were aligned using STAr aligner 2.5.0 (https://github.com/alexdobin/STAR) and counts generated using cufflinks 2.2.1. (http://cole-trapnell-lab.github.io/cufflinks/).

Differentially expressed genes (DEG’s) for the treatment effects were identified based on the counts, using the DESeq2 package in the R programming environment [54,55] (The R Project for Statistical Computing). The scripts used to process and analyze the data, and create the related figures and tables, are found in supplementary methods (Supplementary materials and methods DESeq2). DEG’s for each treatment effect with an absolute log2-fold change of > 0.5 and an adjusted *p*-value of < 0.1 were compared using a Venn diagram. Pheatmap was used to generate heatmaps in R [56]. Expression data including log2-fold change and adjusted *p*-values (method), from the treatment comparisons (G007-LKvsDMSO, GDC-0973vsDMSO and G007-LK/GDC-0973vsDMSO) as well as a list of 2525 genes unique to G007-LK/GDC-0973vsDMSO, were uploaded into Ingenuity Pathway Analysis (IPA) version 01-10 (Qiagen, Redwood City, CA, USA). The expression data, with an absolute log2 fold value of >0.3, >1 or >2, and an adjusted *p*-value of < 0.1 was analyzed using the core analysis function with the Ingenuity Knowledge Base (genes only) reference set and direct relationships, with no filters set for node types, data sources, confidence, species, tissues & cell lines and mutations. Data for predicted canonical pathways and upstream regulators were used with a *z*-score cutoff of > ±0.4 (GDC-0973vsDMSO) or > ±2 (G007-LK/GDC-0973vsDMSO and G007-LK/GDC-0973 unique).

### 4.8. Western Blot Protein Analysis

Cells were lysed in either RIPA buffer (Millipore, Burlington, MA, USA) for total protein extracts or with NE-PER Nuclear and Cytoplasmic Extraction Reagents (Thermo Fisher,) for nuclear and cytoplasmic protein fractions after 24 h of treatment with inhibitors or vehicle control. Equal amounts of protein (15–20 µg) were denatured, separated on SDS-PAGE gels (Bio-Rad Laboratories, Hercules, CA, USA) and transferred to polyvinylidene-difluoride-membranes (Millipore). After blocking in 5% skim milk (AppliChem)/TBS-T for 30 min, membranes were probed with primary antibody in TBS-T over night at 4 °C. Following secondary antibody incubation, proteins were visualized with chemiluminescent substrate (ECL prime Western Blotting Detection Reagent, Sigma-Aldrich). The following primary antibodies were used: AXIN1 (Cell Signalling Technology, Danvers, MA, USA, [CS]#2087), non-phospho (active)-β-catenin (CS#8814), YAP (Santa Cruz Biotechnology, Santa Cruz CA, USA, sc-101199), P-YAP (ser127) (CS#4911), FOXM1 (Santa Cruz Biotechnology, sc-271746), total β-catenin (BD Transduction Laboratories, BD Biosciences, CA, USA, 610153), Tankyrase 1/2 (E10) (Santa Cruz Biotechnology, sc-365897), MEK1/2 (CS#9122), P-MEK1/2 (ser217/221) (CS#9121), GAPDH (Santa Cruz Biotechnology, sc-32233), LAMIN B1 (ab16048-100, Abcam,), ACTIN (A2066, Sigma). Primary antibodies were visualized with secondary IgG-HRP conjugated antibodies (715-035-150 [mouse] or 711-035-152 [rabbit], Jackson ImmunoResearch, West Grove, PA, USA) and enhanced with chemiluminescent substrate (ECL prime Western Blotting Detection Reagent, Sigma-Aldrich, RPN2236). Figures show representative data. ACTIN, GAPDH or LAMINB1 documents equal protein loading. Loading controls were always run on the same blot used for the experimental samples.

### 4.9. Statistical Analysis

All statistical analyses were performed in Excel. For comparisons of two groups, equal variances within the datasets were first analyzed using F-Test. When the F-Test passed (*p* ≥ 0.05), a 2-tailed type 2 Student’s *t*-test was performed. If the F-Test failed (*p* ≤ 0.05), a 2-tailed type 3 Student’s *t*-test was applied. When performing Students *t*-test, *p* < 0.05 was regarded as a statistically significant difference.

## 5. Conclusions

Together these results suggest that combined TNKS/MEK inhibition induces metabolic and oxidative stress responses in HCT-15 cells which promote a positive FOXO3/FOXM1 ratio that synergistically reduces cell growth through molecular characteristics of cell cycle arrest and apoptosis. However, although TNKS inhibition counteracts the MEKi induced feedback rescue mechanism through YAP, cell growth was not entirely eradicated, proposing further rescue mechanisms to maintain cell growth. MEK inhibition has previously been shown to suppress a negative feedback mediated by ERK on HER2/EGFR [57], which is supported by induced activation of EGFR upon MEK inhibition in HCT-15 cells [14], and suggests the need for further inhibitor treatments to overcome rescue mechanisms in this *APC* and *KRAS* mutated cell line.

## Supplementary Materials

The following are available online at http://www.mdpi.com/2072-6694/11/2/164/s1, Figure S1: Biotarget specific responses to TNKS and MEK inhibition, Figure S2: MEK inhibition potentiates HCT-15 cells for growth inhibition by the TNKS inhibitor, Figure S3: Induced canonical WNT signaling upon MEK inhibition is a biotarget specific response, Figure S4: There is a high degree of reproducibility between biological RNAseq replicates, Figure S5: Transcriptional changes of canonical WNT and MEK/ERK target genes upon single and combined TNKSi/MEKi treatments in HCT-15 cells, Figure S6: Molecular and cellular functions associated with the 50 most regulated genes across all treatments in HCT-15 cells, Figure S7: Canonical pathways and molecular functions affected by single TNKSi and MEKi treatments in HCT-15 cells, Figure S8: Characterization of genes regulated by combined TNKSi/MEKi treatment, Figure S9: Molecular and cellular functions affected by combined TNKSi/MEKi treatment, Figure S10: TNKS inhibition reduced transcription of central canonical WNT pathway mediators, Figure S11: TNKS inhibition induced transcription of RAS and PI3K/AKT regulated genes, Figure S12: MEK inhibition reduced transcription of genes involved in cell cycle regulation, Figure S13: MEK inhibition induced transcription of *RAS*, *PI3K* and *ERK1/2*, Figure S14: MEK inhibition enhanced transcription of central canonical WNT pathway mediators, Figure S15: DESeq2 analysis points out FOXM1 and YAP as potential candidates responsible for MEKi induced canonical WNT/β-catenin signaling in HCT-15 cells, Figure S16: MEK inhibition induced hippo/YAP signaling in HCT-15 cells, Figure S17: Combined TNKS/MEK inhibition counteracts the induced MEKi induced hippo/YAP signaling in HCT-15 cells, Figure S18: Transcriptional effects of TNKS inhibition on central survival pathways, Figure S19: Transcriptional effects of MEK inhibition on central survival pathways, Figure S20: Transcriptional effects of combined TNKS/MEK inhibition on central survival pathways, Table S1: Canonical pathways affected by top 50 DEG’s according to IPA, Table S2: IPA predicted upstream regulators of the top 50 DEG’s, Table S3: IPA predicted upstream regulators of group 1 and 3 from the top 50 DEG’s, Table S4: IPA predicted upstream regulators of group 2 from the top 50 DEG’s, Table S5: Canonical pathways affected by tankyrase inhibition according to IPA, Table S6: Upstream regulators affected by tankyrase inhibition according to IPA, Table S7: Canonical pathways affected by MEK inhibition according to IPA, Table S8: Upstream regulators affected by MEK inhibition according to IPA, Table S9: Canonical pathways affected by combined tankyrase and MEK inhibition according to IPA, Table S10: Upstream regulators affected by combined tankyrase and MEK inhibition according to IPA, Table S11: Canonical pathways affected by genes uniquely regulated by combined tankyrase and MEK inhibition according to IPA, Table S12: Upstream regulators affected by genes uniquely regulated by combined tankyrase and MEK inhibition according to IPA, Table S13: DESeq2 analysis showing transcriptional changes induced by tankyrase inhibition compared to DMSO, Table S14: DESeq2 analysis showing transcriptional changes induced by MEK inhibition compared to DMSO, Table S15: DESeq2 analysis showing transcriptional changes induced by combined tankyrase and MEK inhibition compared to DMSO. Supplementary materials and methods DESeq2.
